# Kocuria rosea Sepsis in an Immunocompromised Patient: A Case Report

**DOI:** 10.7759/cureus.68418

**Published:** 2024-09-01

**Authors:** Shanmuga Leela A, Jaya Lakshmi S S, Leela K V, Tanuj M Lamech, Mathew Gerry George, Jayaprakash V

**Affiliations:** 1 Department of Microbiology, SRM Medical College Hospital and Research Centre, Faculty of Medicine and Health Sciences, SRM Institute of Science and Technology, Chengalpattu, IND; 2 Department of Nephrology, SRM Medical College Hospital and Research Centre, Faculty of Medicine and Health Sciences, SRM Institute of Science and Technology, Chengalpattu, IND

**Keywords:** central line-associated bloodstream infection (clabsi), antimicrobial susceptibility testing, chronic kidney disease (ckd), kocuria spp, sepsis

## Abstract

*Kocuria* species, classified within the phylum* Actinomycetota*, class *Actinomycetes*, order *Micrococcales,* family *Micrococcaceae*, are Gram-positive coccoid bacteria. They bear morphological resemblance to *Staphylococci spp *and *Micrococci spp,* which often leads to misidentification and oversight as contaminants, given their presence as normal flora on human and animal skin and mucous membranes. Accurate identification of these organisms typically relies on automated systems such as MALDI-TOF-MS, Vitek-2 System, and 16S rRNA studies. Once considered rare, there is increasing recognition of Kocuria spp due to its emerging involvement in human infections. With increasing reports of infections associated with these bacteria, it is essential for clinical microbiologists to analyze and document the properties of the organism. This will aid clinicians in enhancing patient care and management.

We present the case of a 53-year-old male patient with a complex medical history, including end-stage renal disease on maintenance hemodialysis, anemia of chronic disease, type 2 diabetes mellitus, and a history of rheumatic heart disease status post mitral valve replacement. This patient developed *Kocuria rosea* sepsis secondary to a central line catheter infection, highlighting the emerging clinical significance of *Kocuria* species in immunocompromised individuals.

## Introduction

*Kocuria spp* bacteria are Gram-positive, coccoid organisms belonging to the family *Micrococcaceae* within the phylum *Actinomycetota* [1]. They are typically found in the environment and are known to colonize the skin and mucous membranes of humans and animals, where they are considered part of the normal flora. Initially regarded as contaminants when isolated from clinical specimens due to their similarity in morphology to other bacteria like *Staphylococci spp* and *Micrococci spp*, *Kocuria *species have gained recognition as potential pathogens, particularly in immunocompromised individuals or those with indwelling medical devices [2].

Microbiological identification

*Kocuria* species are non-motile, non-spore-forming, and aerobic bacteria. These bacteria typically possess a rigid cell wall and generally appear Gram-positive, although they may exhibit Gram-variable staining characteristics [3,4]. They thrive aerobically or facultatively anaerobically and prefer neutral pH conditions. They are catalase-positive and coagulase-negative. They stain Gram-positive and appear as cocci arranged in tetrads or clusters [5]. These characteristics can lead to misidentification in clinical microbiology laboratories, often requiring advanced techniques such as MALDI-TOF MS, Vitek systems, or molecular methods like 16S rRNA sequencing for accurate species determination [6].

Clinical significance

While traditionally considered harmless commensals, *Kocuria *species have been increasingly implicated in human infections. These infections can include bacteremia, septicemia, endocarditis, peritonitis, urinary tract infections, and infections associated with indwelling medical devices such as central venous catheters and prosthetic joints [7,8]. Immunocompromised patients, those undergoing invasive medical procedures, or individuals with underlying chronic diseases such as chronic kidney disease or diabetes mellitus are particularly susceptible [9].

Treatment and antimicrobial resistance

The antibiotic susceptibility patterns of *Kocuria* species can vary, making treatment challenging. They are generally susceptible to several antibiotics commonly used to treat Gram-positive bacteria, such as vancomycin, linezolid, and beta-lactams. However, cases of resistance to multiple antibiotics have been reported, necessitating careful consideration of antibiotic choice based on susceptibility testing [10].

## Case presentation

A 53-year-old diabetic male, with a case of end-stage renal disease (ESRD) on maintenance hemodialysis presented with fever and myalgia in the nephrology department. The patient had a significant history of rheumatic heart disease and mitral valve replacement ten years ago. On evaluation, he was conscious, oriented, and febrile with a Fahrenheit temperature of 101.2 and stable vitals. A systemic examination revealed no abnormalities. Owing to pyrexia, elevated INR, and the need for further dialysis, he was admitted to the nephrology ward. Due to persistent fever, which was aggravated during hemodialysis, catheter-related bloodstream infection was suspected, and blood cultures were drawn from the peripheral line and also from the right internal jugular vein catheter site, and the patient was empirically started on cefoperazone with sulbactam and vancomycin in renal doses. A complete hemogram (Table 1) revealed a hemoglobin of 8.2 mg/dL with a PCV of 27%, a white blood cell count of 8310 cells/cu. mm, and a platelet count of 262,000/cu.mm.

**Table 1:** Blood investigations RBC: Red blood cell; PCV: packed cell volume; MCV: mean corpuscular volume; MCH: mean corpuscular hemoglobin; MCHC: mean corpuscular hemoglobin concentration

Test Name	Result	Range	Unit
Complete Hemogram			
Hemoglobin	8.2*	Male: 13 - 17	g/dL
Total RBC Count	3.4	Male: 4.5 - 5.5	million/cu.mm
PCV	27	Male: 40 - 50	%
MCV	77	83 - 101	fL
MCH	24	27 - 32	pg
MCHC	31	31.5 - 34.5	g/dL
Total WBC Count	8310	4000 - 11000	/cu.mm
Platelet Count	262000	150000 - 410000	/cu.mm
Differential Count			
Neutrophils	65.3	40 - 80	%
Lymphocytes	26.2	20 - 40	%
Eosinophils	4	1 - 4	%
Basophils	0.5	0 - 2	%
Monocytes	4	2 - 10	%

During the ICU stay, he developed pyrexia despite the use of optimized antibiotics, such as cefoperazone/sulbactam 1.5g IV Q12th hourly and vancomycin 15 mg/kg IV Q12th hourly. Despite continuous antipyretic support and meropenem injection 500 mg IV Q8th hourly, the patient developed fever spikes associated with chills and rigor. Repeat blood cultures were drawn from the left femoral dialysis catheter and central lines were sent for culture and susceptibility.

**Figure 1:**
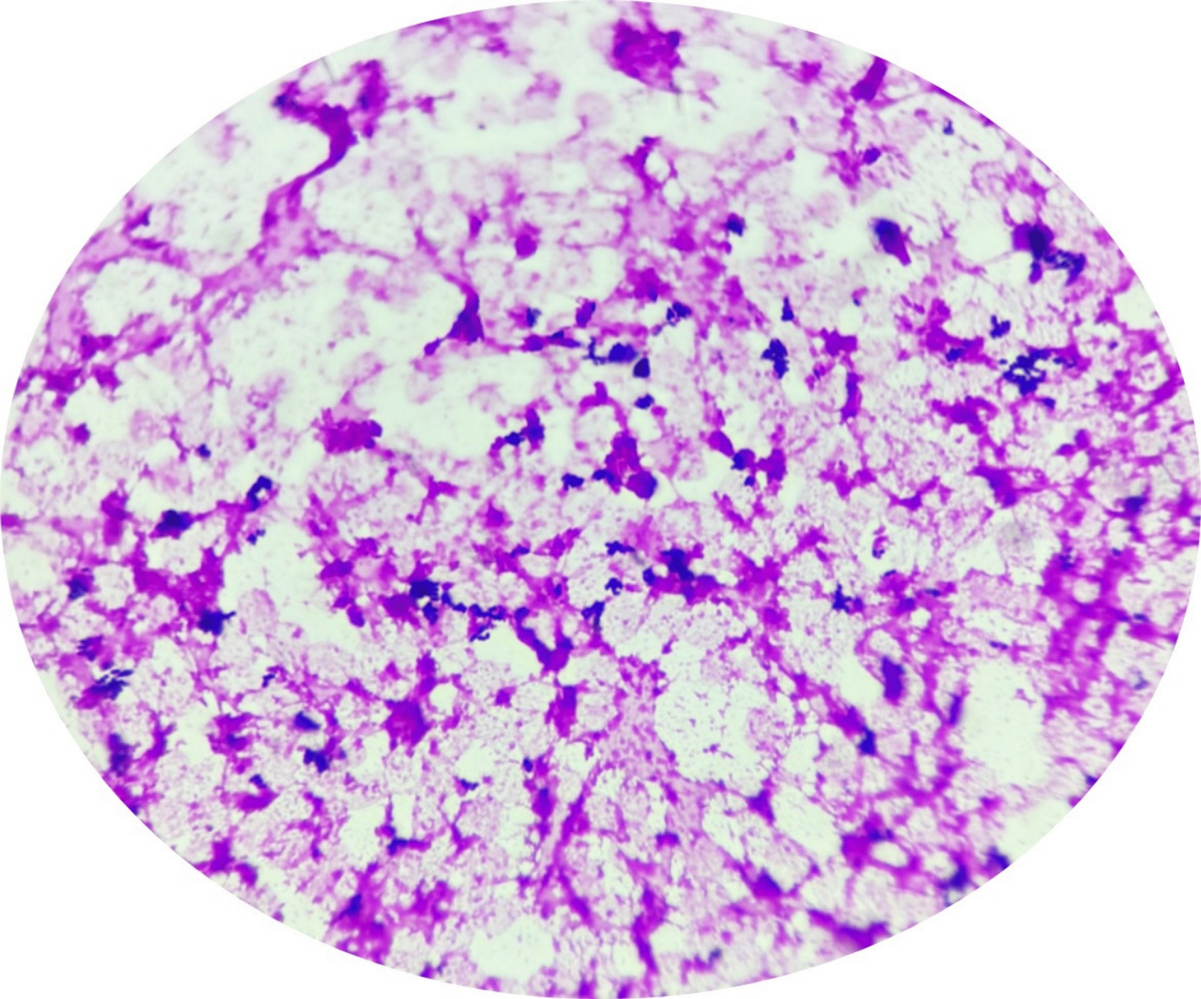
Direct microscopy (Gram stain): Gram-positive spherical cocci in pairs, tetrads, and groups

The blood culture bottle from the central line flagged a positive signal on day 3 of incubation in the BACT/ALERT 3D - bioMérieux India. Direct microscopy of the blood culture bottle revealed Gram-positive spherical cocci in pairs, groups, and tetrads (Figure 1). 

**Figure 2:**
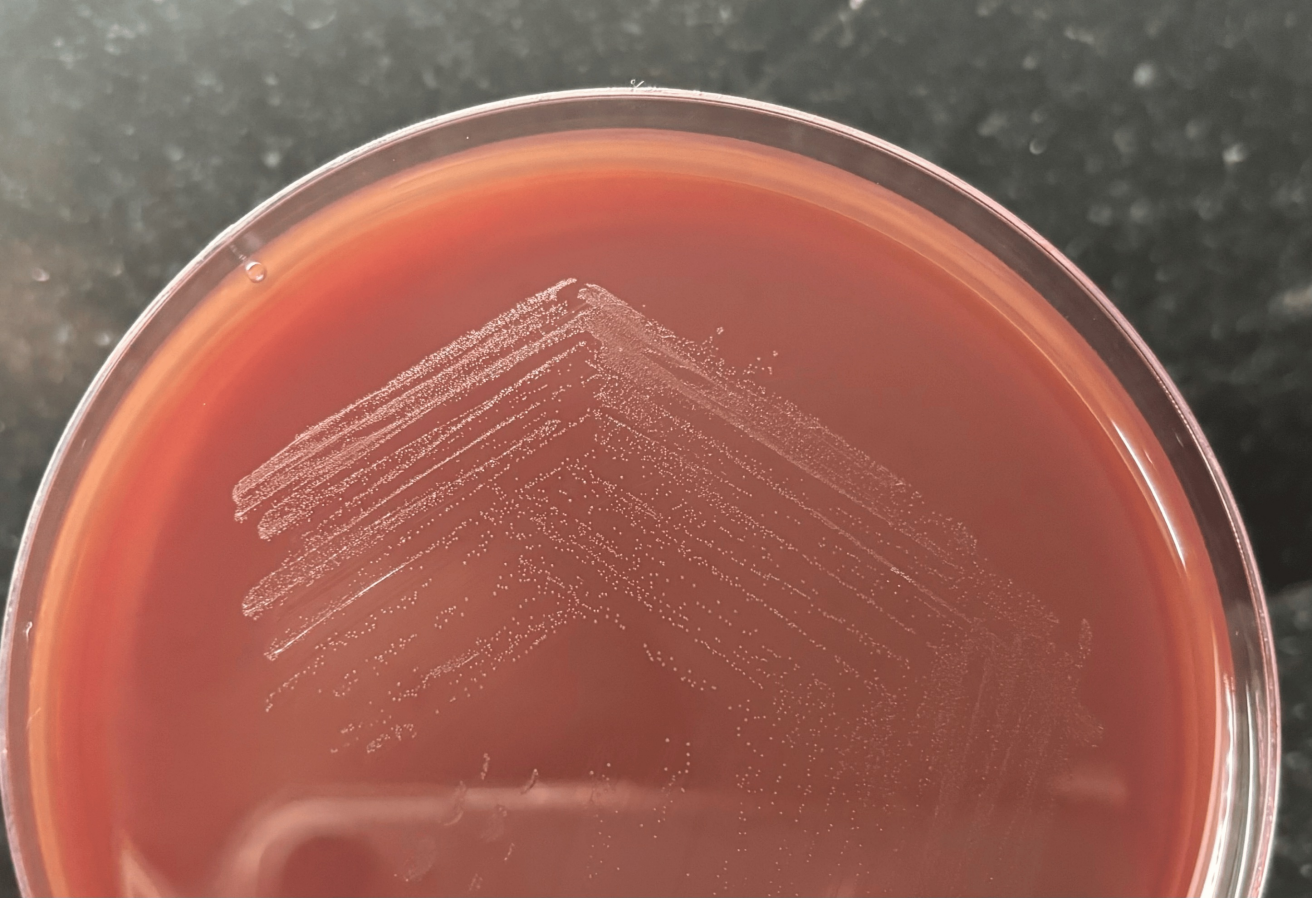
Blood agar shows non-hemolytic, 1-2 mm whitish, small, round, raised, convex, powdery colonies on 24 hours of aerobic incubation

On blood agar, it showed non-hemolytic, 1-2 mm whitish, small, round, raised, convex, powdery colonies on 24 hours of aerobic incubation at 37 °C (Figure 2). Smears from the plates showed similar Gram-positive diplococci, and some were arranged in tetrads.

**Figure 3:**
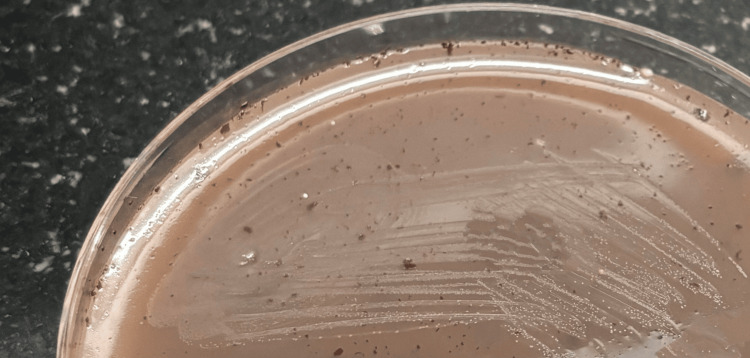
Chocolate agar shows 1-2 mm whitish, small, round, raised, convex, powdery colonies on 24 hours of aerobic incubation

On chocolate agar, there were 1-2 mm whitish, small, round, raised, convex, powdery colonies on 24 hours of aerobic incubation at 37 °C (Figure 3). Smears from the plates showed similar Gram-positive diplococci, and some were arranged in tetrads.

Further characterization using an automated system, VITEK 2 COMPACT (BioMerieux Inc., Durham, NC, USA) identified the organism as *Kocuria rosea, *with the following antimicrobial susceptibility testing (Table 2).

**Table 2:** Antimicrobial susceptibility testing for Kocuria rosea mm: millimeter; DD: disk diffusion method

S.No	Antibiotics	CLSI (Clinical & Laboratory Standards Institute) Guidelines 2024 – Disk Diffusion (DD) method			DD Value of Kocuria rosea	Interpretation
		Susceptible (mm)	Intermediate (mm)	Resistant (mm)		
1.	Azithromycin	> 18	14 - 17	< 13	6	Resistant
2.	Cefepime	> 25	-	< 19	12	Resistant
3.	Ceftriaxone	> 25	-	< 19	6	Resistant
4.	Chloramphenicol	> 18	13 - 17	< 12	30	Susceptible
5.	Clindamycin	> 21	15 - 20	< 14	6	Resistant
6.	Erythromycin	> 18	14 - 17	< 13	6	Resistant
7.	Levofloxacin	> 21	16 - 20	< 15	12	Resistant
8.	Linezolid	> 26	23 - 25	< 22	35	Susceptible
9.	Ofloxacin	> 18	15 - 17	< 14	6	Resistant
10.	Penicillin G	> 29	-	< 28	6	Resistant
11.	Tetracycline	> 19	15 - 18	< 14	33	Susceptible
12.	Vancomycin	> 17	-	< 1	24	Susceptible

The patient was started on linezolid 600mg IV Q12th hourly, as it was one of the susceptible antibiotics of *Kocuria rosea *and the central line was removed. The patient improved clinically and was discharged home with oral linezolid 400mg twice daily for a period of 14 days. During the follow-up visit of the patient, the blood culture was sterile. 

## Discussion

*Kocuria* species, traditionally regarded as harmless skin flora, are now increasingly recognized as potential pathogens in immunocompromised hosts, especially in the context of indwelling medical devices. It belongs to the phylum *Actinomycetota*, Class *Actinomycetes*, Order *Micrococcales*, Family *Micrococcaceae*, Genus *Kocuria* and it has 35 species [11]. The current list of* Kocuria *species is shown in Table 3.

**Table 3:** Current species list of Kocuria spp Source: [11]

Kocuria aegyptia	Kocuria indica	Kocuria salina
Kocuria arsenatis	Kocuria koreensis	Kocuria salsicia
Kocuria assamensis	Kocuria kristinae	Kocuria sediminis
Kocuria atrinae	Kocuria marina	Kocuria soli
Kocuria carniphila	Kocuria massiliensis	Kocuria subflava
Kocuria coralli	Kocuria oceani	Kocuria turfanensis
Kocuria dechangensis	Kocuria ocularis	Kocuria tytonicola
Kocuria erythromyxa	Kocuria palustris	Kocuria tytonis
Kocuria flava	Kocuria pelophila	Kocuria uropygialis
Kocuria gwangalliensis	Kocuria polaris	Kocuria uropygioeca
Kocuria halotolerans	Kocuria rhizophila	Kocuria varians
Kocuria himachalensis	Kocuria rosea	

This case report highlights the crucial role of accurate microbiological identification and targeted antimicrobial therapy in managing such infections. In this case, we encountered* Kocuria rosea* causing sepsis associated with central line-associated bloodstream infection (CLABSI) in an immunocompromised patient. Other reported instances have documented Kocuria spp. causing bacteremia, sepsis, peritonitis, catheter-related infections, infective endocarditis, keratitis, endophthalmitis, and urinary tract infections in both immunocompetent and immunosuppressed patients [12]. The increasing identification of *Kocuria* species in recent times largely relies on automated microbiology systems such as MALDI-TOF, VITEK 2, API, BD Phoenix™, and 16S rRNA studies [13].

Due to limited availability and cost constraints associated with these techniques, infections caused by *Kocuria* species often go unrecognized, misidentified as* Micrococcus spp* contamination, or are dismissed from further identification. However, *Kocuria spp* can be differentiated from *Staphylococci spp* and *Micrococci spp *based on morphological and cultural characteristics, as well as differential antimicrobial susceptibility testing [14]. On culture plates,* Kocuria spp *typically forms small, non-hemolytic colonies around 1-2 mm in diameter on blood agar, with prolonged incubation yielding mild yellow pigmentation. Gram staining reveals a Gram-positive diplococci, tetrads, and group arrangement in our isolated species, which typically show susceptibility to bacitracin and lysozyme, while displaying resistance to nitrofurantoin, furazolidone, and lysostaphin. Biochemically, these bacteria exhibit significant variability, as they respond differently to standard laboratory identification tests, such as catalase, urease, and citrate utilization tests. Typically, they do not ferment mannitol and lack both bound and free coagulase enzymes. Additionally, a modified oxidase test helps differentiate Kocuria species (which are oxidase negative) from Micrococci. 

Among the 35 species of *Kocuria, Kocuria kristinae *and *Kocuria rosea* are most commonly associated with infections such as infective endocarditis, synovitis, pneumonia, and sepsis. *Kocuria rhizophila *has also been reported to cause relapsing peritonitis in patients undergoing peritoneal dialysis [15]. In this specific case, *Kocuria rosea* was isolated from a sepsis patient, where the central line was identified as the source of CLABSI. The patient, immunosuppressed and undergoing regular hemodialysis, developed significant sepsis but recovered completely upon initiation of appropriate antibiotics. Similar case reports have highlighted Kocuria's role in causing CLABSI, facilitated by its commensal nature on the skin, facilitating entry into systemic sites.

Currently, there are no established susceptibility testing guidelines or reference ranges for *Kocuria* species under CLSI or EUCAST, necessitating reliance on MIC or disk diffusion zone size criteria established for Staphylococcal or Streptococcal species. Generally resistant to cephalosporins, *Kocuria* species tend to be sensitive to antibiotics like penicillin, tetracycline, vancomycin, and linezolid, with sporadic reports of susceptibility to gentamicin, amikacin, tobramycin, and teicoplanin for certain species. The increasing prevalence of Kocuria infections poses a significant challenge to healthcare systems, underscoring the urgent need for further research and guideline development to optimize treatment strategies for infections caused by these organisms [16].

## Conclusions

This case report highlights a rare yet clinically significant occurrence of *Kocuria rosea *sepsis in a patient with ESRD, underscores the complexities involved in diagnosing and treating infections caused by uncommon pathogens, and emphasizes the essential role of multidisciplinary care in achieving favorable outcomes for critically ill patients.

In conclusion, although* Kocuria* bacteria are typically considered part of the normal human microbiota and often disregarded as contaminants in clinical practice, their capacity to cause infections, particularly in immunocompromised individuals, emphasizes the critical need for accurate identification and appropriate management. Ongoing research into their pathogenic mechanisms and resistance patterns is essential to enhance clinical outcomes associated with* Kocuria* infections.
